# Complete Genome of *Bacillus subtilis* Myophage Grass

**DOI:** 10.1128/genomeA.00857-13

**Published:** 2013-12-05

**Authors:** Stanton Y. Miller, Jennifer M. Colquhoun, Abbey L. Perl, Karthik R. Chamakura, Gabriel F. Kuty Everett

**Affiliations:** Center for Phage Technology, Texas A&M University, College Station, Texas, USAa; Department of Biological Sciences, Lehigh University, Bethlehem, Pennsylvania, USAb

## Abstract

*Bacillus subtilis* is a ubiquitous Gram-positive model organism. Here, we describe the complete genome of *B. subtilus* myophage Grass. Aside from genes encoding core proteins pertinent to the life cycle of the phage, Grass has several interesting features, including an FtsK/SpoIIIE protein.

## GENOME ANNOUNCEMENT

*Bacillus subtilis* is a soil-dwelling, endospore-forming, Gram-positive bacterium (1). *B. subtilis* is well studied and has been used in many commercial applications, such as the protection of crop growth and biofilm analysis (2, 3). It is also a well-understood model for determining the properties of spores. The study of *B. subtilis* phages has contributed greatly to our understanding of the genetics and physiology of this model organism. Here, we present the complete annotated genome of the *B. subtilis* phage Grass.

Grass was isolated from a soil sample collected in Bethlehem, PA. Phage DNA was sequenced using 454 pyrosequencing at the Emory GRA Genome Center (Emory University, Atlanta, GA). The trimmed FLX Titanium reads were assembled to a single contig at 128.6-fold coverage using the Newbler assembler version 2.5.3 (454 Life Sciences) with the default settings. The contigs were confirmed to be complete by PCR. Genes were predicted using GeneMarkS (4) and corrected using software tools available on the Center for Phage Technology (CPT) portal (https://cpt.tamu.edu/cpt-software/portal/).

Grass contains a 152,440-bp unique genome with a coding density of 89.1% and a G+C content of 42.3%. In the unit genome, 242 coding sequences were identified, of which 92 were novel hypothetical genes and 95 were conserved hypothetical genes. The genome encodes 3 tRNAs and contains 38 rho-independent terminators. Electron microscopy imaging shows that Grass is a myophage. The TerL showed homology to the TerLs of phages with long terminal repeats. The raw sequencing data were processed using the Pause (https://cpt.tamu.edu/cpt-software/releases/pause/) method to show that the terminal repeat is 4,208 bp in length.

Genes encoding proteins pertaining to replication and recombination, DNA biosynthesis, packaging, morphogenesis, and lysis were identified. Genes for replication and modification proteins include UvsW helicase, DnaB-like helicase, primase, polymerase, RecA, and several DNA-binding proteins and nucleases. Genes encoding DNA biosynthesis proteins include dUTPase, dihydrofolate reductase, thymidylate synthase, nucleotidyltransferase, ribonucleotide diphosphate reductase subunits alpha and beta, and a ribonucleotide reductase stimulatory protein. The large terminase and portal proteins were also annotated. Genes encoding phage morphogenesis proteins were identified (major and minor capsid proteins, tail sheath proteins, tail lysin, baseplate assembly proteins [P2 W {InterPro database no. IPR007048} and P2 J {pfam04865}], and a tailspike protein with a pectin lyase domain). A holin (class II with two transmembrane domains in an N-in C-in topology) and endolysin (*N*-acetylmuramoyl-l-alanine amidase) were also found.

Interesting features in the Grass genome are genes encoding an FtsK/SpoIIIE protein, an RtcB family protein, and a ParM protein. SpoIIIE is an ATPase common in sporulating bacteria that pumps DNA into the forespore (5). RtcB is an RNA ligase involved in tRNA splicing and repair (6). ParM is an actin homolog involved in plasmid segregation that is also found in the lytic *Bacillus* phage SPO1. How these proteins are involved in phage replication and assembly is unknown.

### Nucleotide sequence accession number.

The genome sequence of phage Grass was contributed as accession no. KF669652 to GenBank.
